# Supplementary material for: Place of Residence Is Associated with Dietary Intake and BMI-SDS in Children and Adolescents: Findings from the DONALD Cohort Study
Source: Int J Environ Res Public Health. 2023 Dec 28;21(1):46. doi: 10.3390/ijerph21010046 (PMC10815849; doi:10.3390/ijerph21010046)
Supplement: Supplementary file 1 [file ijerph-21-00046-s001.zip › ijerph-2740223-supplementary.pdf]

Supplementary Materials

Figure S1: Chloropleth maps of mean dietary intake and nutrient status indicators of Donald study participants (age 6-18) between 2014 and 2019 by city districts (Stadtbezirk) of Dortmund

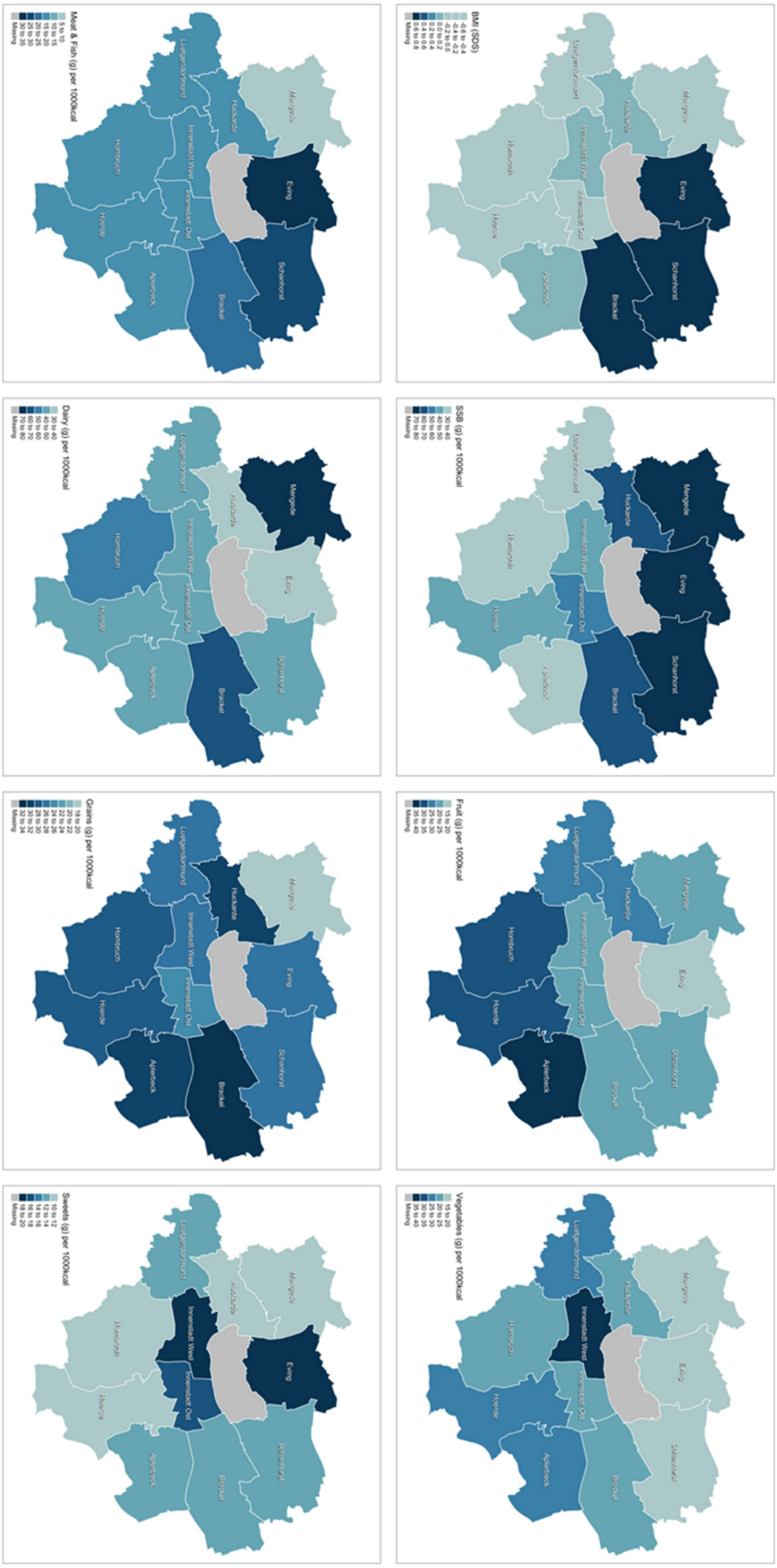

Figure S2: Predicted values for North and South Dortmund, respectively, by model specification

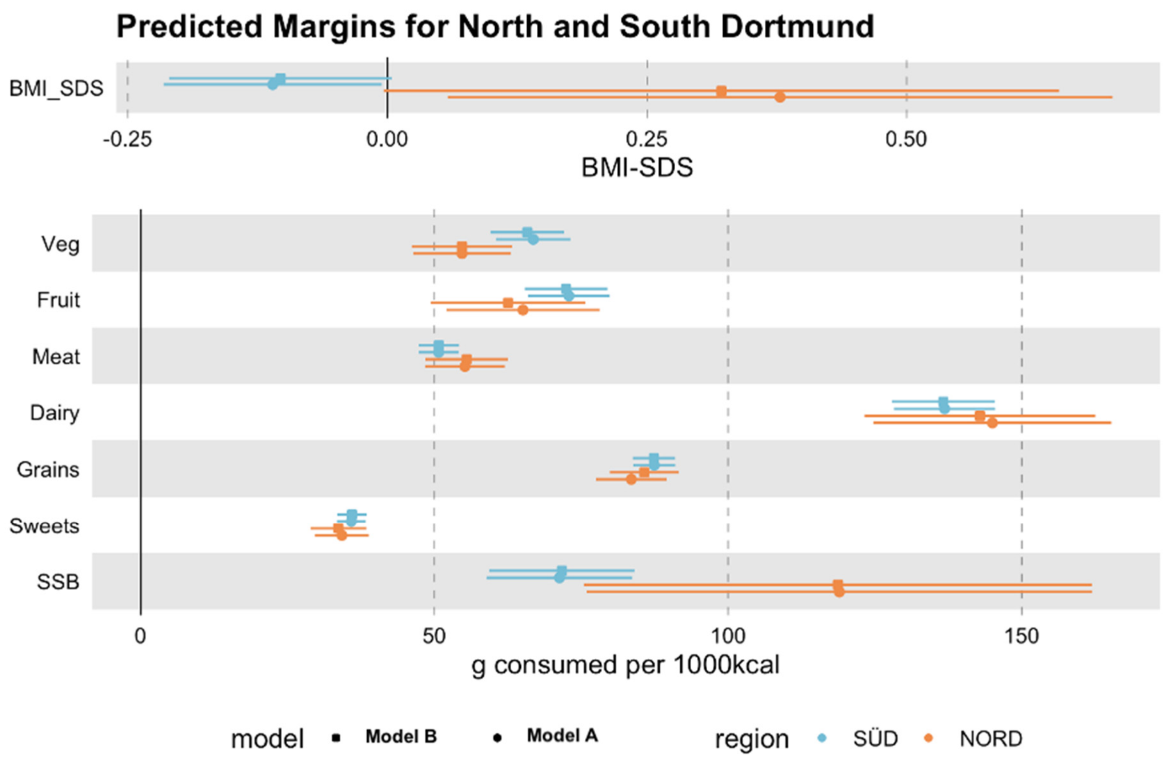

Table S1: Regression results for macronutrients by model specification

			β	p-value	Benjamini-Hochberg p-value	Lower CI	Upper CI
**BMI**	SDS	Model A	-0.489	**0.005**	**0.005**	-0.827	-0.151
		Model B	-0.425	**0.016**	**0.016**	-0.769	-0.081
		Model B*	-0.417	**0.017**	**0.017**	-0.759	-0.075
**Food Groups**	SSB	Model A	-47.661	**0.039**	**0.138**	-92.821	-2.501
		Model B	-47	**0.044**	**0.138**	-92.642	-1.359
	Vegetables	Model A	12.133	**0.027**	**0.135**	1.377	22.889
		Model B	11.129	**0.043**	**0.138**	0.351	21.906
	Fruit	Model A	7.829	0.302	0.539	-7.03	22.688
		Model B	9.876	0.196	0.490	-5.094	24.847
	Meat	Model A	-4.468	0.252	0.525	-12.122	3.185
		Model B	-4.746	0.239	0.525	-12.64	3.148
	Sweets	Model A	1.602	0.545	0.706	-3.592	6.796
		Model B	2.327	0.397	0.662	-3.057	7.712

	Grain	Model A	3.905	0.277	0.533	-3.141	10.952
		Model B	1.646	0.637	0.758	-5.2	8.492
	Dairy	Model A	-8.138	0.465	0.706	-29.946	13.671
		Model B	-6.237	0.565	0.706	-27.492	15.017
	Protein	Model A	-0.001	0.668	0.759	-0.008	0.005
		Model B	-0.002	0.524	0.706	-0.009	0.005
**Macronutrients**	Carbohydrates						
		Model A	-0.001	0.861	0.861	-0.014	0.012
		Model B	-0.002	0.754	0.785	-0.016	0.011
	Fat						
		Model A	0.002	0.723	0.785	-0.011	0.016
		Model B	0.004	0.558	0.706	-0.01	0.018
	Sugar						
		Model A	-0.02	**0.021**	**0.131**	-0.036	-0.003
		Model B	-0.016	0.055	0.153	-0.032	0

Model A is adjusted for age and sex.
Model B is adjusted for age, sex and socio-economic status.
Model B* (BMI-SDS only) is adjusted for age, sex, socio-economic status and physical activity.
FDR (False Discovery Rate) was calculated following Benjamini-Hochberg procedure for each model family and outcome group and set at 0.20.

BMI Body Mass Index, *SDS* Standard Deviation Score*, SSB* Sugar-Sweetened Beverages, *CI* Confidence Intervall, *FDR* False Discovery Rate
